# Size-dependent kinetics during non-equilibrium lithiation of nano-sized zinc ferrite

**DOI:** 10.1038/s41467-018-07831-5

**Published:** 2019-01-09

**Authors:** Jing Li, Qingping Meng, Yiman Zhang, Lele Peng, Guihua Yu, Amy C. Marschilok, Lijun Wu, Dong Su, Kenneth J. Takeuchi, Esther S. Takeuchi, Yimei Zhu, Eric A. Stach

**Affiliations:** 1 Department of Materials Science and Chemical Engineering, SUNY-Stony Brook University, Stony Brook, NY 11794 USA; 2 Department of Condensed Matter Physics, Brookhaven National Laboratory, Upton, NY 11973 USA; 3 Department of Chemistry, SUNY-Stony Brook University, Stony Brook, NY 11794 USA; 4 Materials Science and Engineering Program and Department of Mechanical Engineering, The University of Texas at Austin, Austin, TX 78712 USA; 5 Energy Sciences Directorate, Brookhaven National Laboratory, Interdisciplinary Sciences Building, Building 734, Upton, NY 11973 USA; 6 Center for Functional Nanomaterials, Brookhaven National Laboratory, Upton, NY 11973 USA; 7 Department of Materials Science and Engineering, University of Pennsylvania, Philadelphia, PA 19104 USA

## Abstract

Spinel transition metal oxides (TMOs) have emerged as promising anode materials for lithium-ion batteries. It has been shown that reducing their particle size to nanoscale dimensions benefits overall electrochemical performance. Here, we use in situ transmission electron microscopy to probe the lithiation behavior of spinel ZnFe_2_O_4_ as a function of particle size. We have found that ZnFe_2_O_4_ undergoes an intercalation-to-conversion reaction sequence, with the initial intercalation process being size dependent. Larger ZnFe_2_O_4_ particles (40 nm) follow a two-phase intercalation reaction. In contrast, a solid-solution transformation dominates the early stages of discharge when the particle size is about 6–9 nm. Using a thermodynamic analysis, we find that the size-dependent kinetics originate from the interfacial energy between the two phases. Furthermore, the conversion reaction in both large and small particles favors {111} planes and follows a core-shell reaction mode. These results elucidate the intrinsic mechanism that permits fast reaction kinetics in smaller nanoparticles.

## Abstract (web summary)

Reducing particle size of electrode materials to nanoscale dimensions is believed responsible for their enhanced reaction kinetics and electrochemical performance. Here, the authors use in situ transmission electron microscopy to study the dynamic process of the spinel zinc ferrite nanoparticles as a function of size, finding that the intercalation reaction pathway changes below a critical particle size.

## Introduction

There is an ever-increasing demand for lithium-ion batteries that have both large energy density and improved cycling life. With respect to anode materials, the most competitive candidates are those that exhibit alloying and/or conversion reactions because they can provide a much larger capacity than carbonaceous anode materials. A primary challenge to the adoption of these electrode materials is the huge capacity loss that they experience during cycling, which is generally attributed to the strain induced by the large volume changes that occur during lithiation and delithiation. Prior studies have shown that reducing particle size can help to relax the strain, and that this can lead to improved cyclability^1,2^. Reducing materials to nanoscale dimensions also leads to a high surface-to-volume ratio and reduced transport length^3,4^. Thus, there have been substantial efforts devoted to the development of nano-sized electrode materials^5–14^. Recent studies regarding lithiation of intercalation compounds have indicated that the intrinsic ionic/electronic transport behaviors can be tuned by controlling particle size^15–18^. Kobayashi et al. found that the miscibility gap of LiFePO_4_ cathode materials shrinks as particle size reduces, which results in a homogeneous single-phase transformation pathway for small particles (<100 nm)^19^. To date, a single-phase transformation pathway in nano-sized electrodes has only been observed in LiFePO_4_ and the relationship between particle size and reaction pathway has not been clarified in conversion-type electrode materials.

Spinel transition metal oxides (TMOs) have been shown to be a high specific capacity anode material for lithium-ion batteries^20–22^. Among TMOs, Fe_3_O_4_ has received significant attention due to its nontoxicity, low cost, and high electronic conductivity^23–25^. However, reduced Fe^0^ is not electrochemically active with Li^+^. Compounds in which another electroactive transition metal ion is substituted for one iron atom in the Fe_3_O_4_ structure have recently gained attention^14,26–28^. Zinc ferrite (ZnFe_2_O_4_), for example, has a theoretical capacity of 1000 mAhg^−1^, an improvement over Fe_3_O_4_ that results from the fact that metallic Zn can further alloy with additional Li^+^ after the conversion reaction has occurred^29^. The process of lithium insertion into spinel zinc ferrite ([Zn^2+^]_8a_[Fe^3+^_2_]_16d_O_4_, denoted by the Wyckoff notation) has been studied by Waszczak et al. using X-ray diffraction^30^, and can be expressed as: [Zn^2+^]_8a_[Fe^3+^_2_]_16d_O_4_ + Li^+^ → [Li^+^Zn^2+^]_16c_ [Fe^3+^_2_]_16d_O_4_ + 7Li^+^ → Zn^0^ + Fe^0^ + 4Li_2_O. Previous studies have reported that ZnFe_2_O_4_ powder and thin films suffer poor capacity reversibility and limited capacity when they have a particle size that is of the micron scale^31,32^. A more recent study found that even when the particles have nanoscale dimensions (<15 nm), the rate performance and cyclability of ZnFe_2_O_4_ still varied significantly with particle size^33^. These results indicate that in order to improve electrochemical performance it is crucial to explore the origin of particle size effects on reaction kinetics and elucidate the reaction mechanism.

To this end, we utilize the in situ dry cell transmission electron microscopy (TEM) technique, an approach which allows direct observation of structural changes at the nano- and atomic scale in real time^34–38^. Because of the absence of liquid electrolyte, the dry cell configuration enables high spatial resolution, which in turn allows real-time high-resolution TEM (HRTEM) imaging at atomic resolution. Moreover, because these HRTEM images can be subsequently converted to diffractograms using the Fast Fourier transforms (FFT) technique, in situ HRTEM imaging is capable of visualizing phase evolution and correlating it directly with morphological changes. In this work, we investigate the kinetics of small (6–9 nm) vs. large (ca. 40 nm) ZnFe_2_O_4_ nanoparticles using in situ TEM. Although both the large and small ZnFe_2_O_4_ nanoparticles undergo both an intercalation and a subsequent conversion reaction process, we find that the lithiation pathways of the intercalation process varies with particle size: the small nanoparticles show a solid-solution behavior while the large nanoparticles undergo a two-phase intercalation reaction. We explain the origin of the size-dependent kinetics using a straightforward thermodynamic theory.

## Results

### Materials characterization and electrochemical properties

**Fig. 1:**
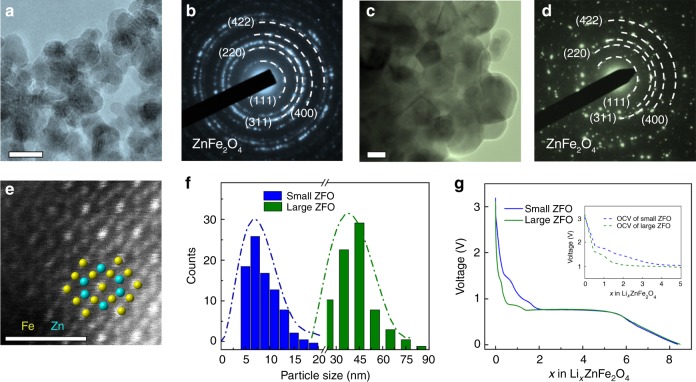
Characterization of pristine materials and electrochemical properties. **a** Typical morphology of S-ZFO (scale bar: 10 nm) and **b** the corresponding SAED pattern. **c** Typical morphology of L-ZFO (scale bar: 10 nm) and **d** the corresponding SAED pattern. **e** HAADF-STEM image showing the spinel structure along the <101> zone axis, compared to an overlaid atomic model along the same projection. Scale bar: 1 nm. **f** Size distribution of S-ZFO (blue) and L-ZFO (green). **g** Discharge profile of S-ZFO (blue) and L-ZFO (green) measured at the rate of 200 mAg^−1^. Inset shows the OCV profile of S-ZFO and L-ZFO, respectively

Zinc ferrite (ZnFe_2_O_4_, abbreviated as ZFO hereafter) nanoparticles with different crystalline sizes were synthesized (Fig. 1). The small ZFO (Fig. 1a) nanoparticles are 6–9 nm in size while the large ZFO (Fig. 1c) are of ~40 nm in size, as illustrated in Fig. 1f. Both the small and large nanoparticles have the spinel structure, with the Fd3̄m Space Group, as confirmed by X-ray diffraction (Supplementary Figure 1) and corresponding selected area electron diffraction (SAED) patterns, shown respectively in Fig. 1b, d. In addition, the overall sample is polycrystalline, with individual particles having high crystallinity, as indicated by the atomic-resolution high-angle annular dark field (HAADF) image shown in Fig. 1e. In order to understand the electrochemical performance of ZFO as a function of particle size, both large and small ZFO were discharged to 0.01 V at a rate of 200 mAg^−1^. Theoretically, ZFO can take up to eight Li^+^ per formula during the conversion reaction. As shown in Fig. 1g, both large and small ZFO receive more than eight Li^+^ at the end of initial discharge: this excess is generally ascribed to the presence of side reactions, electrolyte decomposition and solid-electrolyte interface formation^39^. When fully lithiated, the pristine ZFO, in both cases, has evolved into a nanocomposite composed of ultrafine Fe^0^ and Zn^0^ nanoparticles embedded in a matrix of amorphous Li_2_O: this has been confirmed by ex situ TEM (Supplementary Figures 2 and 3). The overall lithiation reaction is independent of particle size and can be expressed as: ZnFe_2_O_4_ + 8Li^+^ + 8e^−^ → Zn^0^ + 2Fe^0^ + 4Li_2_O. As shown in Fig. 1g, distinct plateaus appear as the lithiation reaction proceeds. Previous studies have assigned each plateau to specific lithiation processes^29,40,41^: (1) Li^+^ initially inserted into 16c sites (~ 1.64 V), (2) further incoming Li^+^ forcing the Zn^2+^ at the 8a site to neighboring empty 16c sites (~ 1.58 V), and (3) the generation of the final discharge product (<1 V). The major difference in the kinetic response of the small ZFO (S-ZFO) and large ZFO (L-ZFO) occurs at the lower depth of discharge (DOD), which is associated with the Li^+^ intercalation process. To investigate the mass transfer in detail, we utilized a galvanostatic intermittent titration technique (GITT) test at a rate of 62.5 mAg^−1^ to measure the open-circuit voltage (OCV) at different DOD, as depicted in the inset of Fig. 1g and Supplementary Figure 4. The OCV reflects the redox potential at the equilibrium state, whereby L-ZFO undergoes a two-step reaction with the formation of an intermediate phase. In contrast, the smooth decrease of the OCV in S-ZFO suggests the existence of a continuous, homogeneous single phase. However, every electrochemical reaction happens at a certain overpotential: thus, thermodynamics is not the only factor that controls the reaction pathway. In order to achieve an accurate understanding of the lithiation pathways, we have utilized in situ electron microscopy techniques to probe the reaction kinetics during operation^42,43^.

### Phase evolution investigated by in situ electron diffraction

**Fig. 2:**
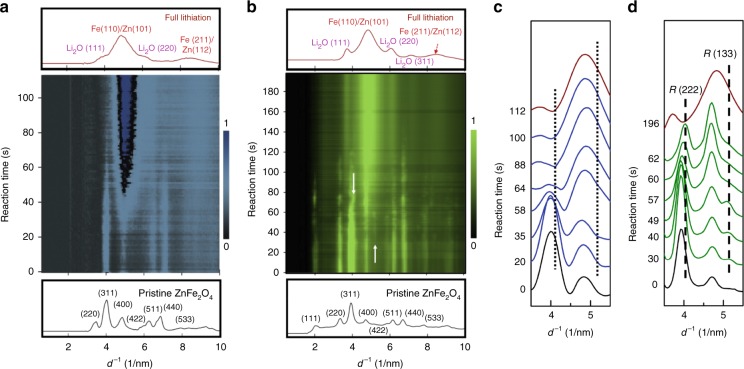
Phase evolution tracked by in situ electron diffraction. Electron diffraction intensity profiles of **a** S-ZFO and **b** L-ZFO as a function of reaction time. The corresponding radial intensity spectrum of pristine (black) and fully lithiated (red) states are shown below and above the color map, respectively. **c** Radially integrated intensity profiles of S-ZFO as function of reaction time. The dotted lines point out the corresponding d-spacing of ordered rock-salt phase observed in **d**. **d** Radially integrated intensity profiles of L-ZFO as a function of time. The additional phase indicated by white arrows in **b** corresponds to the ordered rock-salt phase (*R* (222) and *R* (133)), as indicated by black dashed lines

We utilize in situ electron diffraction to probe the phase evolution of both samples throughout the entire lithiation process. In Fig. 2, we plot the radially integrated intensity profiles of S-ZFO and L-ZFO as a function of reaction time, respectively, as extracted from their corresponding time-sequenced SAED patterns (see also Supplementary Figure 5 and Supplementary Movie 1 and 2). In the pristine state, the 111 peak of S-ZFO is missing after background subtraction (as shown in the bottom of Fig. 2a). This is because the 111 peak is too dim and close to the transmitted spot, which overwhelms the signal from it. For better visualization, we false-color the intensity profile (see Supplementary Figure 5) of S-ZFO with blue (Fig. 2a) and L-ZFO with green (Fig. 2b). For both samples, the initial sharp Bragg reflections associated with spinel ZFO (bottom spectrum) gradually change with lithiation into broadened peaks associated with Li_2_O and Zn^0^/ Fe^0^ (top spectrum). This is the same as is observed in the ex situ data, which validates the approach. It is worth noting that two additional peaks appear at the lower DOD for the L-ZFO (<60 s), indicated by the white arrows in Fig. 2b. For clearer visualization, we extract and display the time-sequential intensity profiles of L-ZFO in the range where the new phase occurs, as shown in Fig. 2d. Comparing with the pristine state (black curve) and the fully lithiated state (red curve), it can be seen that two distinct peaks emerge at 4.04 nm^−1^ and 5.08 nm^−1^, which are associated with 222 and 133 reflections of the ordered rock-salt phase ([Li_*x*_^+^ Zn^2+^]_16c_[Fe^3+2]^_16d_O_4_, denoted by the Wyckoff notation). These are indicated by the black dashed line in Fig. 2d. The formation of the ordered rock-salt phase can be ascribed to the process of incoming Li^+^ repelling [Zn^2+^]_8a_ into neighboring empty 16c sites. The structure of the ordered rock-salt phase is equivalent to a standard rock-salt structure, where both kinds of cations equally occupy all of the 16c sites. Upon further lithiation, both peaks gradually decrease in intensity with the emergence of the Li_2_O 111 and Fe 110 / Zn 101 reflections, thereby indicating that material has undergone the conversion process. In summary, L-ZFO undergoes a two-step reaction during lithiation (intercalation, then conversion) whereby the ordered rock-salt intermediate phase is first formed, then subsequently decomposed. Intriguingly, in the S-ZFO, the additional reflections found at 4.04 nm^−1^ and 5.08 nm^−1^ in the L-ZFO are not present, as shown by the dotted lines in Fig. 2c. As the lithiation reaction proceeds in S-ZFO, the intensity of the Bragg reflections associating with the spinel phase monotonically decreases with increasing intensity of the reflections associated with Fe/Zn and Li_2_O phases, indicating that the S-ZFO exhibits a “direct” conversion upon lithiation (Supplementary Figure 6).

### Two-step reaction pathway in L-ZFO visualized by in situ HRTEM

**Fig. 3:**
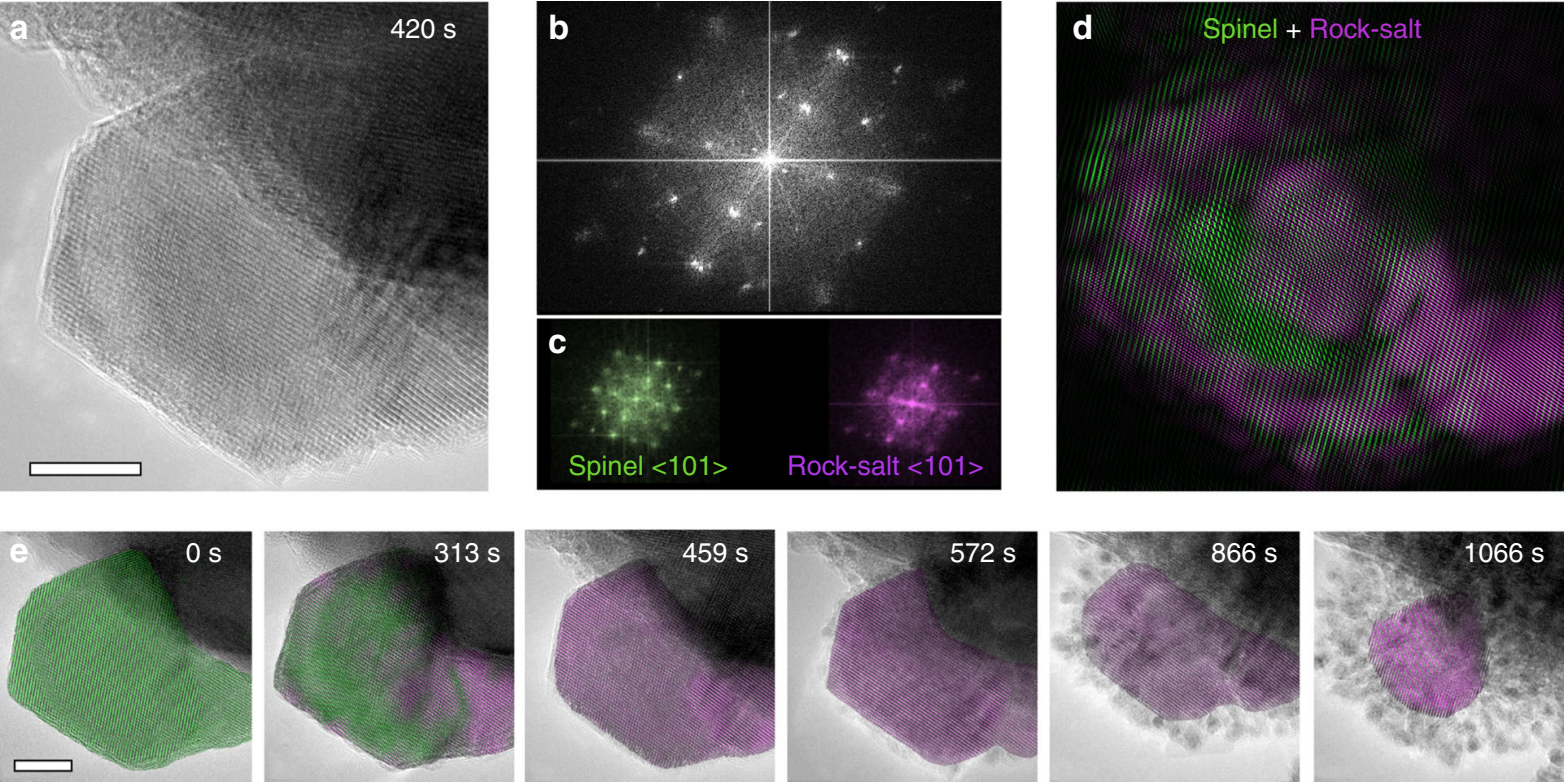
Two-phase transformation mechanism probed via in situ HRTEM imaging. **a** HRTEM image of partially reacted L-ZFO obtained at 420 s (scale bar: 10 nm) and **b** the corresponding FFT pattern. **c** Two sets of FFT patterns extracted from **b** representing coexistence of spinel (green) and ordered rock-salt phase (magenta). **d** Inverse FFT image shows the distribution of spinel (green) and ordered rock-salt phase (magenta) in real space. **e** Time-sequenced HRTEM images with phase information (false-color) showing the transformation pathway of L-ZFO as a function of reaction time. Scale bar: 10 nm

In order to obtain a more comprehensive understanding of the lithiation process, the microstructural evolution of L-ZFO during lithiation is investigated in real space (Supplementary Movie 3). One general deficiency of in situ techniques is the inability to obtain information in both real space and reciprocal space at the same time^44–48^. However, local microstructural changes and phase evolution can be achieved quasi-simultaneously via the use of in situ HRTEM, followed by subsequent use of FFT. Using this approach, detailed information is accessible at different DOD (see detailed phase analysis in Supplementary Information). For example, the corresponding FFT pattern (Fig. 3b) is produced along with the partially lithiated particle (Fig. 3a). The FFT pattern includes two sets of diffraction spots, which are associated with the spinel structure and ordered rock-salt structure, respectively (Fig. 3c). Using these two set of spots in the FFT, we can map the distribution of the spinel (green) and ordered rock-salt (magenta) phase within a single nanoparticle (Fig. 3d) by inverting the FFT with only this information preserved^49^. Similarly, by combining the time-sequenced phase distribution maps with the corresponding HRTEM images, as shown in Fig. 3e (Supplementary Figure 7), we can track the evolution of both the phase information and the morphological information at the same time, within individual nanoparticles. We see here that the lithiation process is driven by Li^+^ diffusion from the bottom of the image to the top. At the initial stage of discharge (<492 s), incoming Li^+^ ions push [Zn^2+^]_8a_ to nearby empty 16c sites, leading to a phase transformation from the spinel (green false color) to the ordered rock-salt phase (magenta false color). Further lithiation triggers the subsequent conversion reaction, during which the intermediate phase evolves into the final discharge products of ultra-small metallic Zn and Fe nanoparticles and lithium oxide. In addition, the conversion reaction is observed to generally propagate from the exterior surface (along {111} planes) toward the particle center, whereas no preferential lithiation pathway is observed during the initial intercalation process. Based on observations in both real space and reciprocal space, we can conclude that the L-ZFO undergoes a two-step reaction, which initiates with random Li^+^ intercalation, followed by a “core-shell” pathway upon the subsequent conversion reaction.

### Solid-solution transformation in S-ZFO visualized by in situ HRTEM

**Fig. 4:**
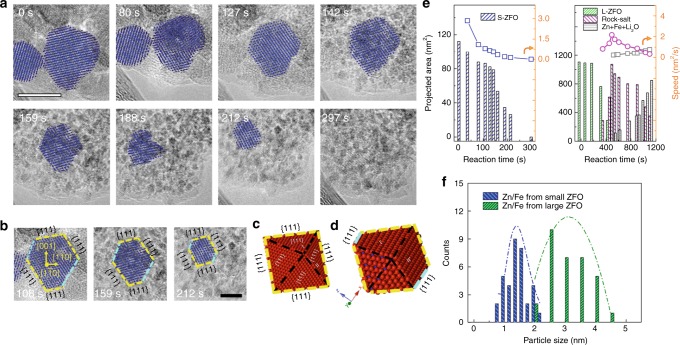
In situ HRTEM study of S-ZFO and comparison of reaction kinetics of ZFO with different particle size. **a** Time-sequenced HRTEM images with phase information (overlaid false color) showing the phase evolution of S-ZFO as a function of reaction time. Scale bar: 10 nm. **b** Enlarged HRTEM images illustrating the preferred reaction interface. **c** Atomic model of {111} octahedron viewing along <110>direction. Scale bar: 5 nm. **d** Atomic model showing the structure of partially reacted {111} octahedron. **e** Projected area of spinel phase in S-ZFO (left panel, blue) and the three phases occurring in L-ZFO (right panel, green: L-ZFO, magenta: ordered rock-salt and gray: nanocomposite) changes as a function of time. Curves on the top of each panel showing the propagation speed of each reaction, which is the derivative of the projected area of each phase to reaction time (vertical axis on the right). **f** Size distribution of final discharge products (Zn^0^/Fe^0^) generated from S-ZFO (blue) and L-ZFO (green), respectively

Similarly, we investigated the lithiation of S-ZFO in real time (Supplementary Movie 4). Unlike the two-phase reaction process observed in L-ZFO, S-ZFO exhibits a solid-solution transformation during lithiation, as shown in Fig. 4a. The S-ZFO nanoparticle retains the spinel structure (blue) until the completion of the conversion reaction, which is in agreement with previous in situ electron diffraction results (see Fig. 2a, c and detailed analysis in Supplementary Figure 6). In addition, we found the orientation of the particle changes during our in situ TEM observation. As shown in Fig. 4b (Supplementary Figure 8), the orientation of that particle changed to the <110> zone axis at 108 s from the <111> zone axis. It is interesting to note that by 108 s the exposed facets are {111} planes and these facets were maintained until the particle was fully lithiated (297 s), as indicated by the yellow and turquoise dashed lines in Fig. 4b. The presence of preferential exposed {111} facets is also observed in other S-ZFO particles (Supplementary Figure 9). An atomic model in Fig. 4c represents the projection of a ZFO with {111} planes as the exposed facets, viewed along a <110> direction. The model structures are rotated to match the experimental data and slightly tilted to permit visualization of the three-dimensional out-of-plane features. In projection, the partially reacted particle (Fig. 4b) is a truncated {111} octahedron with two vertexes decomposed into a nanocomposite. Based on previous studies, the reconstructed {111} surface planes are more stable than the {100} surface planes, and it is preferable to have exposed facets that have more transition metal cations^50–52^. With respect to ZFO, the {111} planes of ZFO have one more Fe^3+^ than the {100} planes, suggesting a faster Fe^3+^/Fe^0^ redox reaction on the {111} plane. This would lead to the formation of the “cropped” vertices (shown as the turquoise dashed line), which is where the intersection {111} planes occurs. This broadly verifies that the {111} planes are the most preferred reaction interface, as depicted in Fig. 4d.

With the assumption that the electrochemical condition within one single particle is identical, we measured the reaction kinetics of S-ZFO and L-ZFO, respectively, by quantifying the projected areas of each phase within individual particles as a function of reaction time (Fig. 4e). The propagation speed of the solid-solution reaction is found to be no greater than 3 nm^2^/s. For the L-ZFO, the projected areas of three phases—spinel, ordered rock-salt, and nanocomposite—are measured, respectively. The propagation speed of the two-phase intercalation reaction is found to be one order of magnitude faster than the subsequent conversion reaction. In addition, S-ZFO generates finer metallic nanoparticles than those found in the nanocomposite created from L-ZFO (Fig. 4f). The presence of ultrafine-sized metallic Fe and Zn would further reduce the Li^+^ diffusion length in subsequent cycles, which may lead to enhanced rate capability for electrode materials with small particle size.

## Discussion

**Fig. 5:**
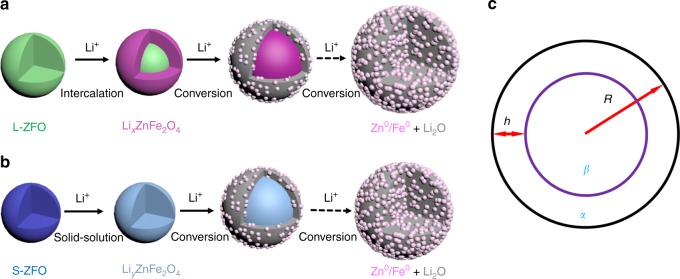
Phase transformation pathway of L-ZFO and S-ZFO nanoparticles. Schematic 3D models illustrating the lithiation pathway of **a** L-ZFO and **b** S-ZFO. **c** Schematic diagram shows the coexistence of pre-existing phase (*β*) and newly formed phase (*α*) inside a particle undergoing a core-shell reaction mode, where *R* is the size of the particle and *h* is the thickness of *α* phase coated on the *β* phase

The aforementioned lithiation pathways of S-ZFO and L-ZFO are schematically illustrated in Fig. 5a, b, respectively. We found that L-ZFO undergoes a two-step reaction, in a manner similar to many other spinel TMOs^24,30,53^. The reaction mechanism can be expressed as:

1 $$\left[ {{\mathrm{Zn}}^{2 + }} \right]_{8{\mathrm{a}}}\left[ {{\mathrm{Fe}}_2^{3 + }} \right]_{16{\mathrm{d}}}{\mathrm{O}}_4 \to \left[ {{\mathrm{Li}}_x^ + {\mathrm{Zn}}^{2 + }} \right]_{16{\mathrm{c}}}\left[ {{\mathrm{Fe}}_2^{3 + }} \right]_{16{\mathrm{d}}}{\mathrm{O}}_4 \to {\mathrm{Zn}}^0 \\ + \, {\mathrm{Fe}}^0 + {\mathrm{Li}}_2{\mathrm{O}}.$$

In addition, we conducted ex situ HRTEM imaging on ZFO at an early DOD (*x* < 1). A similar mixture of spinel and rock-salt phases was found in ex situ sample (Supplementary Fig. 10): this further demonstrates consistency between the in situ and ex situ results. Intriguingly, the S-ZFO does not follow the two-phase intercalation reaction pathway. The primary features of the two-phase intercalation reaction—the formation of the ordered rock-salt intermediate phase and the corresponding volume change (~4.4%)—are not observed in S-ZFO upon lithiation. The negligible volume expansion we see in S-ZFO was also corroborated at the macroscopic scale via the in situ XRD technique^41^. Our results strongly suggest that S-ZFO follows a solid-solution transformation pathway, leading to a solid-solution phase (Li_*y*_ZnFe_2_O_4_) as the initial state of lithiation, as depicted in Fig. 5b.

The differences between the intercalation pathways of the large and small ZFO may originate from the difference in interfacial energy between the pre-existing phase and the new phase. These concepts can be explored within a well-established thermodynamic theory: the phase separation from a homogenous concentration *x*_*ε*_ to a mixture of *α* phase with concentration *x*_*α*_ and *β* phase with concentration *x*_*β*_ can happen only when the change of Gibbs free energy is negative^54^. The change of Gibbs free energy (Δ*G*) can be expressed as Eq. (2):

2 $${{\Delta G}} = {{V}}_a{{g}}_a(x_a) + {{V}}_{{\beta }}{{g}}_{{\beta }}(x_{{\beta }}) + S_{{\alpha }}\sigma _{{\alpha }} + S_{{\beta }}\sigma _{{\beta }} + S_{{{\alpha \beta }}}{{\gamma }} - {{Vg}}_{{\varepsilon }}(x_{{\varepsilon }}).$$

where the *V* is the volume of the crystallite, *g*_*n*_(*x*_*n*_) is the free energy of the phase per unit volume, *x*_*n*_ is the concentration of solute atoms of each phase, *S* is the surface area, *σ* is surface energy and *γ* is the energy of the interface between the *α* and *β* phases, assuming the surface energy and the volume of a particle remain the same during the phase transformation (*ε* → *α* + *β*). Conventionally, the surface and interfacial energy terms in Eq. (2) do not contribute significantly since the particle size is large. However, as the particle size decreases, the contribution of the interfacial energy is no longer negligible. This is because the driving force for the transformation scales with volume, while the interfacial energy—which hinders the transformation—scales with area. Based on our in situ observations, we can see that the phase transformation of ZFO proceeds via a core-shell mode (Fig. 5c). Thus, the associated change of Gibbs free energy is as follows:

3 $${{\Delta G}} = \frac{{4\pi }}{3}\left[ {{{R}}^3-\left( {{{R}}-h} \right)^3} \right]{{g}}_{{\alpha }}(x_{{\alpha }}) + \frac{{4\pi }}{3}\left( {{{R}}-h} \right)^3{{g}}_{{\beta }}(x_{{\beta }}) \\ + \, 4\pi \left( {{{R}}-h} \right)^2{{\gamma }}-\frac{{4\pi }}{3}{{R}}^3{{g}}_{{\varepsilon }}(x_{{\varepsilon }})$$

in which

4 $$h = {{R}}-{{R}}\left( {\frac{{x_{{\alpha }} - x_{{\varepsilon }}}}{{x_{{\alpha }} - x_{{\beta }}}}} \right)^{\frac{1}{3}},$$

as determined by the lever rule. From Eqs. (3) and (4), we find the critical particle size to be:

5 $${{R}} \ge \frac{{3{{\gamma }}\left( {\frac{{x_{{\alpha }}-x_{{\varepsilon }}}}{{x_{{\alpha }}-x_{{\beta }}}}} \right)^{\frac{2}{3}}}}{{{{\Delta }}g_{\mathrm v}}}$$

that leads to the phase separation (Δ*G* ≤ 0), where ∆*g*_v_ is the change of free energy per unit volume after the phase transformation. In other words, particles less than the critical size $\left( {R^{\ast} = \frac{{3\gamma\left( {\frac{{x_{{\alpha }}-x_{{\varepsilon }}}}{{x_{{\alpha }}-x_{{\beta }}}}} \right)^{\frac{2}{3}}}}{{{{\Delta }}g_{\mathrm v}}}} \right)$ will undergo a solid-solution transformation, without the formation of the ordered rock-salt Li_*x*_ZnFe_2_O_4_. This is consistent with our in situ TEM observations of the S-ZFO particles, where we only observed a direct conversion process. For the solid-solution process, the reaction kinetics are not limited by the migration speed of phase boundary: this fact may benefit the cycling performance. It is also worth noting that analysis above regarding the size-dependent interfacial energy can be extended to other intercalation oxide electrode materials. Furthermore, during the conversion process, we observed a core-shell reaction mode. The volume expansion induced by the conversion reaction (the shell) produces a compressive strain on the core which can retard further reaction^55^. In other words, the smaller particle size ZFO particles are, the less compressive strain they suffer. Therefore, during both intercalation and conversion processes, the reaction kinetics are enhanced by small particle sizes.

In summary, we have investigated the lithiation pathway of spinel zinc ferrite as a function of particle size using in situ TEM techniques. We have found that below a critical size (9 nm < R* <40 nm) the two-phase reaction pathway is suppressed and a solid-solution process dominates the intercalation reaction, which is analogous to the case of LiFePO_4_. Moreover, we have found that the enhanced electrochemical performance of small electrode materials is not only a result of the reduced Li^+^ and e^−^ diffusion length but also benefits from the modification of both the electrochemical reaction pathway and the reduction of the strain during the reaction. In addition, we found the subsequent conversion reaction prefers to proceed via {111} planes regardless of particle size: this indicates that crystallographic structure also strongly impacts electrochemical performance and Li^+^ transport. These results suggest a rational approach for improving the electrochemical performance of other conversion-type electrode materials and provide a criterion for the design and selection of spinel TMOs as anode materials with enhanced rate capability and cycling life.

## Methods

### Sample preparation

ZnFe_2_O_4_ nanomaterials were prepared using a coprecipitation method. Briefly, stoichiometric solutions of Zn- and Fe-based nitrate salts (Zn(NO_3_)_2_, Fe(NO_3_)_3_) were added concurrently to a deionized water solution containing excess triethylamine in an ice bath. The precipitate was collected and vacuum-dried. For small ZnFe_2_O_4_, the precipitate was treated hydrothermally at 120 °C for 12 h using DI water as solvent. The small ZnFe_2_O_4_ was further prepared by using graphene oxide as a template and Pluronic copolymers with 4400 molecular weight as surfactants. The large ZnFe_2_O_4_ sample is obtained by heat treatment of the precipitate at 500 °C for 6 h. The final sample is washed and vacuum-dried.

### Electrochemical measurements

The composite electrode used for electrochemical measurements were prepared with 80 wt% active material, 10 wt% carbon black, and 10 wt% polyvinylidene fluoride in *N*-methyl-2-pyrrolidone and cast onto a copper foil current collector. R2032-type coin cells were assembled inside an argon-filled glove box with the as-prepared composite electrode as cathode and Li metal as anode. A Celgard 2400 monolayer polyethylene separator and 1 M lithium hexafluorophosphate (LiPF_6_) solution in ethylene carbonate:dimethyl carbonate (DMC) (1:1 in weight) were used as the electrolyte for coin cells. Battery testing was performed using a battery test station (Arbin BT2000) at room temperature. Each current pulse performed during the GITT measurements was followed by a 24-h relaxation period to ensure full relaxation of the OCV.

### TEM characterization

The in situ dry cell was assembled into a Nanofactory STM specimen holder inside an argon-filled glove box. The electrochemical cell is composed of three parts: (1) metallic Li that is coated on a piezo-driven tungsten tip that functions as an anode, (2) Li_2_O formed on the surface of Li anode which functions as a solid electrolyte and (3) ZnFe_2_O_4_ powder dispersed on a half TEM grid with amorphous carbon support work which functions as the positive electrode. After installation, the holder is transferred to the TEM column within a sealed, argon-filled bag in order to avoid air exposure. During operation, a constant negative DC potential was applied between the positive electrode and the Li source in a range of 3~5 V (discharge). The lithiation process was observed in real time by TEM imaging or diffraction mode. The ex situ samples after cycling were directly removed from the coin cell to a DMC solution inside an argon-filled glove box. For sample preparation, the cycled materials were sonicated and dispersed on a TEM grid. In situ and ex situ TEM characterization were done on JEOL JEM-2100F transmission electron microscope equipped with a field-emission electron gun that operated at 200 kV. Analytical EELS and high-resolution HAADF imaging were performed at a Hitachi-2700C scanning transmission electron microscope operated at 200 kV, which is equipped with cold field-emission gun and a probe aberration corrector, yielding a spatial and energy resolution down to 1 Å and 0.35 eV, respectively.

## Supplementary Information

Supplementary Information

Description of Additional Supplementary Files

Supplementary Movie 1

Supplementary Movie 2

Supplementary Movie 3

Supplementary Movie 4

## Data Availability

All data generated or analyzed during this study are included in this published Article and its Supplementary Information files. Further information is also available from the corresponding author upon reasonable request.
