# Supplementary material for: Size-dependent kinetics during non-equilibrium lithiation of nano-sized zinc ferrite
Source: Nat Commun. 2019 Jan 9;10:93. doi: 10.1038/s41467-018-07831-5 (PMC6327060; doi:10.1038/s41467-018-07831-5)
Supplement: Supplementary file 1 — Supplementary Information [file 41467_2018_7831_MOESM1_ESM.pdf]

Size-Dependent Kinetics during Non-Equilibrium Lithiation of Nano-sized Zinc Ferrite

Li *et al.*

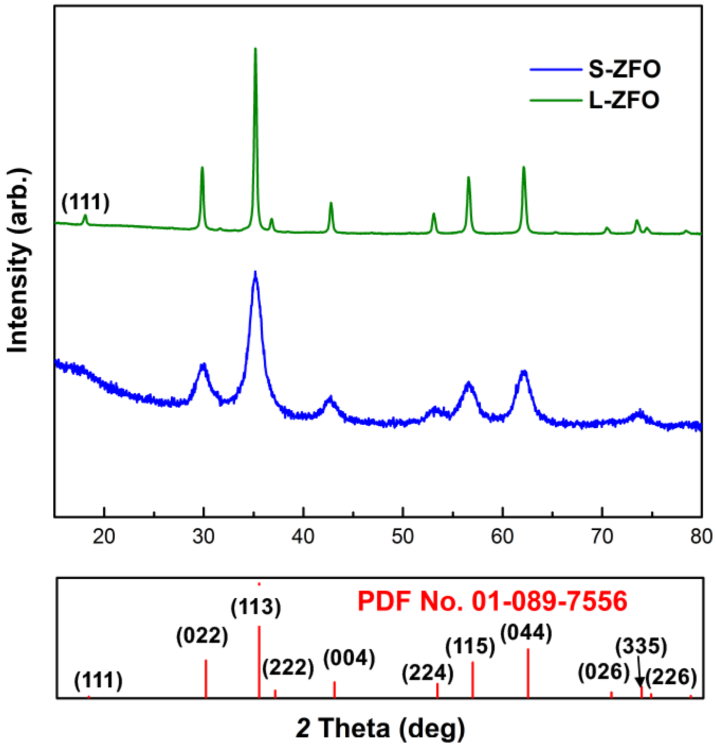

Supplementary Figure 1. XRD patterns of the as-synthesized S-ZFO (blue) and L-ZFO (green) compared to reference pattern of spinel $ZnFe_2O_4$ (PDF No. 01-089-7556).

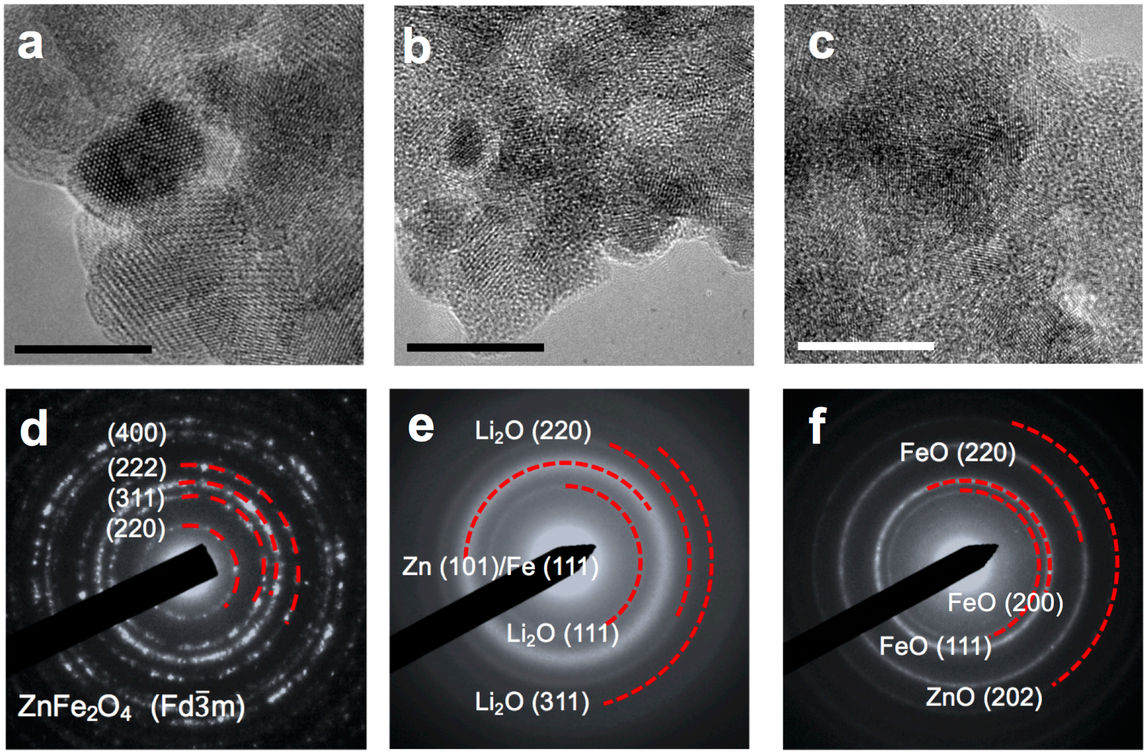

Supplementary Figure2. *Ex situ* TEM images and SAED patterns showing typical morphologies and corresponding phase information of S-ZFO at: (a), (d) pristine state; (b), (e) discharge state (0.01V); and (c), (f) charge state (3.0V). Scale bars: 10 nm.

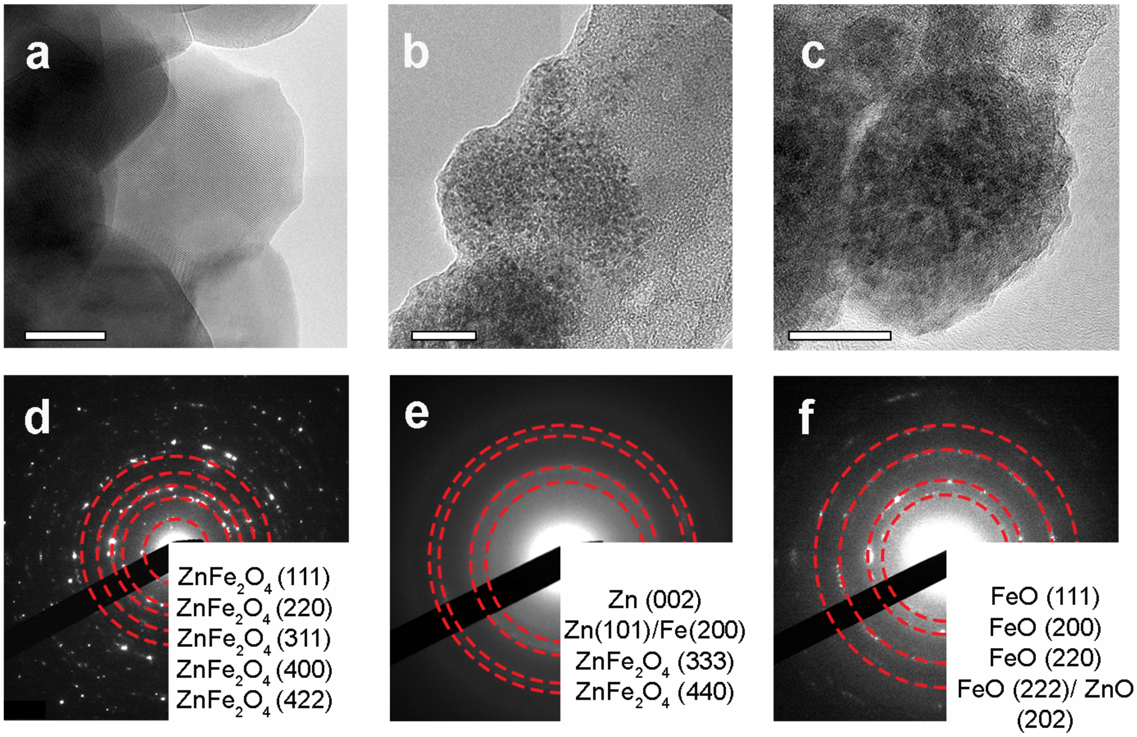

Supplementary Figure3. *Ex situ* TEM images and SAED patterns showing typical morphologies and corresponding phase information of L-ZFO at: (a), (d) pristine state; (b), (e) discharge state (0.01V); and (c), (f) charge state (3.0V). Scale bars: 20 nm.

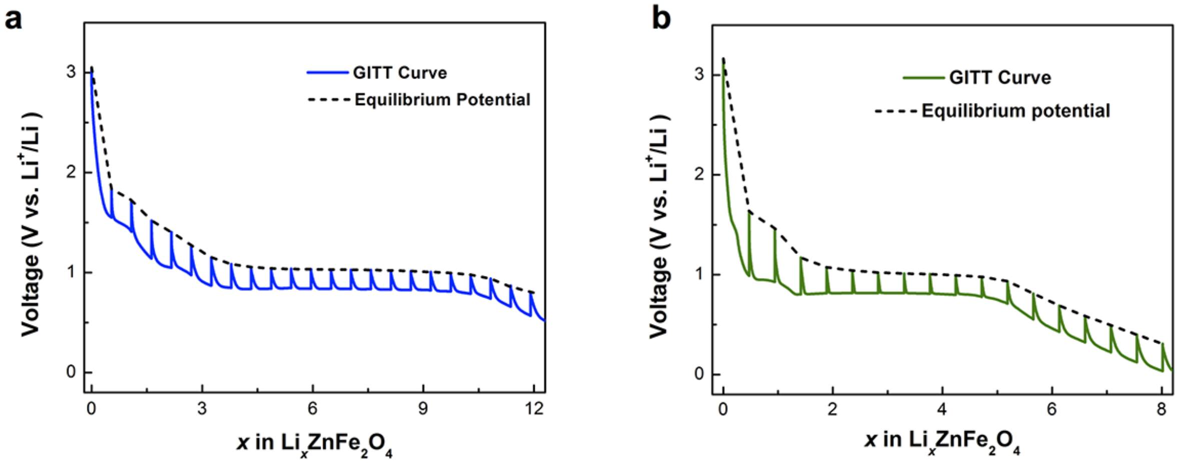

Supplementary Figure 4. GITT and OCV profiles of (a) S-ZFO and (b) L-ZFO during the first discharge, measured at 62.5 mAg^{-1}.

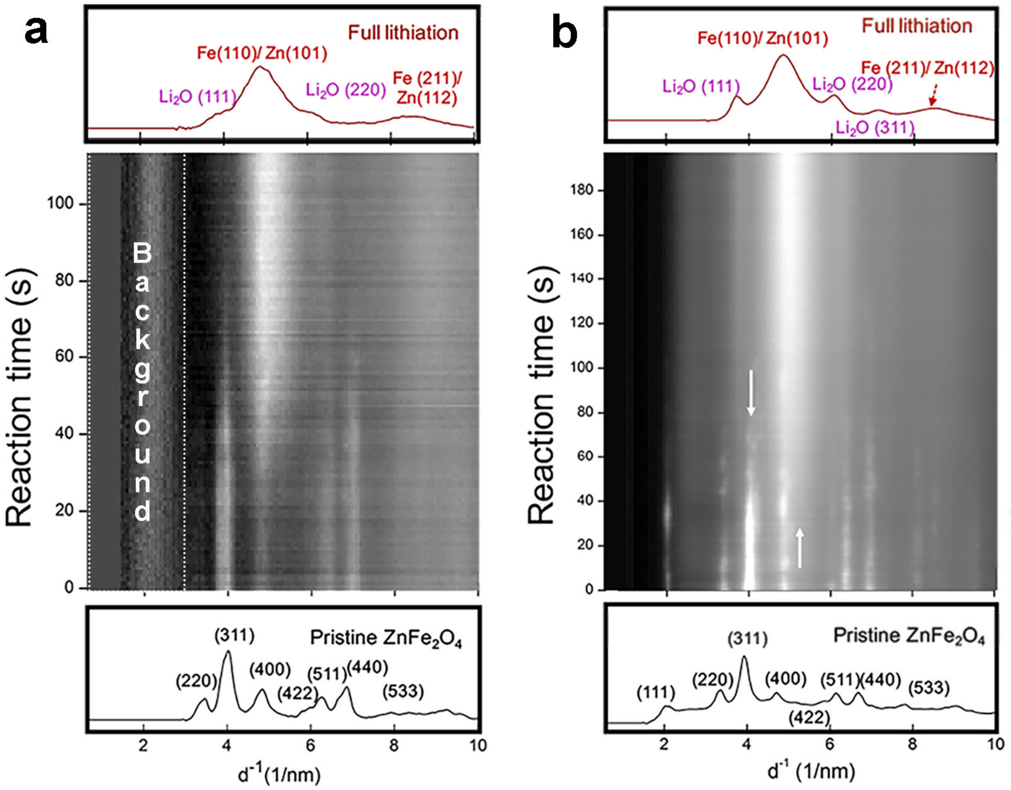

Supplementary Figure 5. Electron diffraction intensity profiles of **(a)** S-ZFO and **(b)** L-ZFO as a function of reaction time plotted in greyscale.

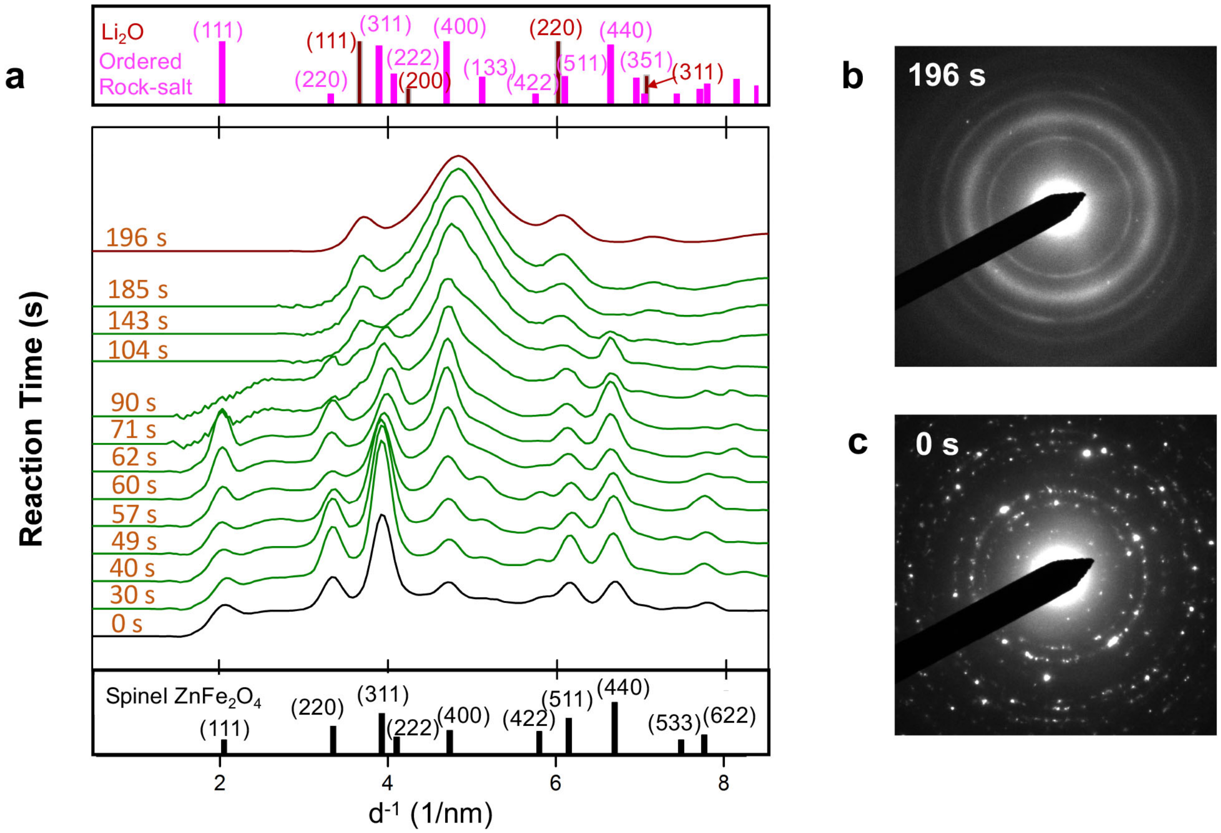

Supplementary Figure 6. (a) Radial integrated intensity profiles plotted as a function of reaction time showing phase evolution of L-ZFO upon lithiation. Corresponding references of pristine spinel structure(black), intermediate ordered rock-salt(magenta) structure and fully lithiated Li_2O (red) are listed below and above the spectrum, respectively. (b) SAED pattern of L-ZFO after lithiation (196 s). (c) SAED pattern of L-ZFO at pristine state (0 s).

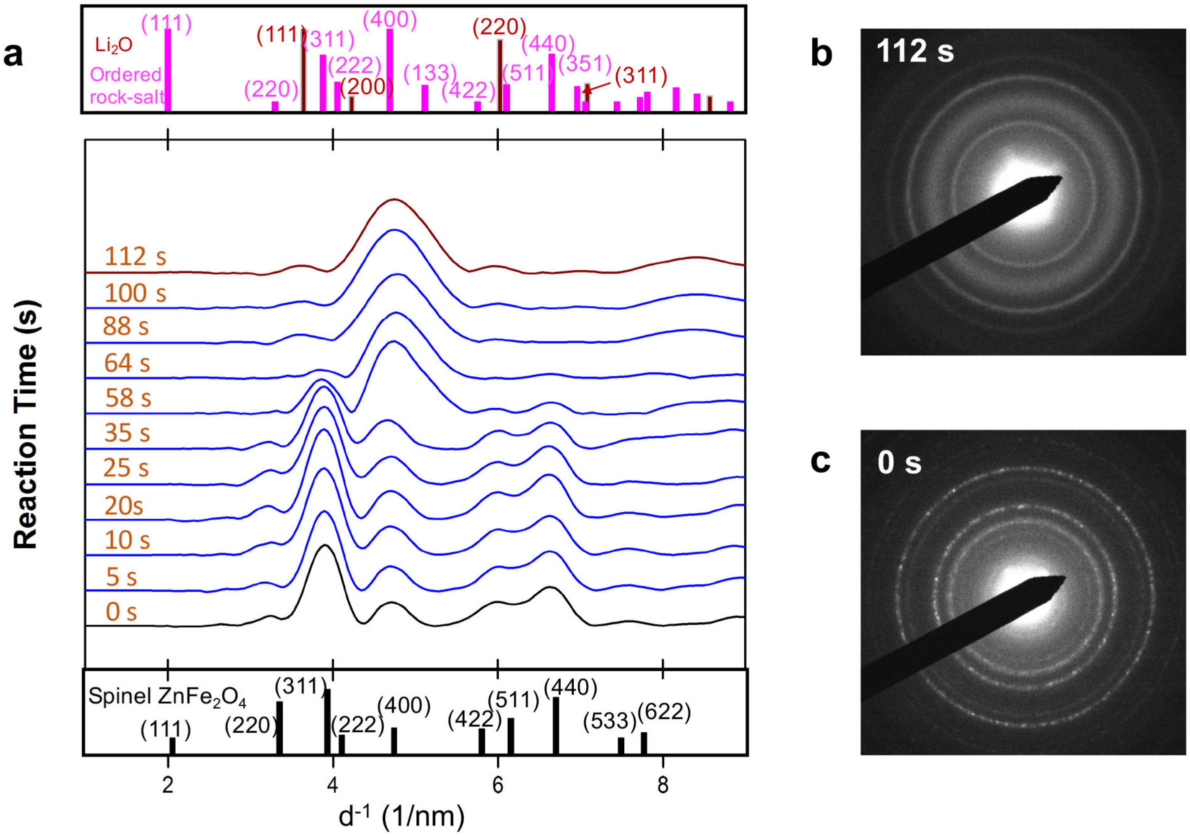

Supplementary Figure 7. (a) Radial integrated intensity profiles plotted as a function of time showing phase evolution of S-ZFO upon lithiation. Corresponding references of pristine spinel structure (black), intermediate ordered rock-salt (magenta) structure and fully lithiated Li_2O (red) are listed below and above the spectrum, respectively. (b) SAED pattern of S-ZFO after lithiation(112 s). (c) SAED pattern of S-ZFO at pristine state (0 s).

Fast Fourier Transform (FFT) and Inverse FFT analyses. FFT pattern is directly generated from the HRTEM images, which is able to show the difference of the closely related phases in localized areas. Two sets of diffraction patterns were obtained respectively by using live FFT with a small selected area (128 X 128 pixels), as shown in Supplementary Figure 5a. After characterization, those two sets of diffraction spots are found associated to spinel (green) and ordered rock-salt structure (magenta) along <101> zone axis, which are in agreement of simulated diffraction pattern (Supplementary Figure 5c). Putting mask on characterization spots of each set of diffraction patterns and keep the masked area with edge smoothen by 5 pixels, inverse FFT

images showing the corresponding phase information can be achieved, as shown in Supplementary Figure 5d. The FFT patterns and corresponding inverse FFT images are generated by using Gatan DigitalMicrograph® software. The simulation of SAED patterns are obtained by SingleCrystal software from CrystalMaker ® software.

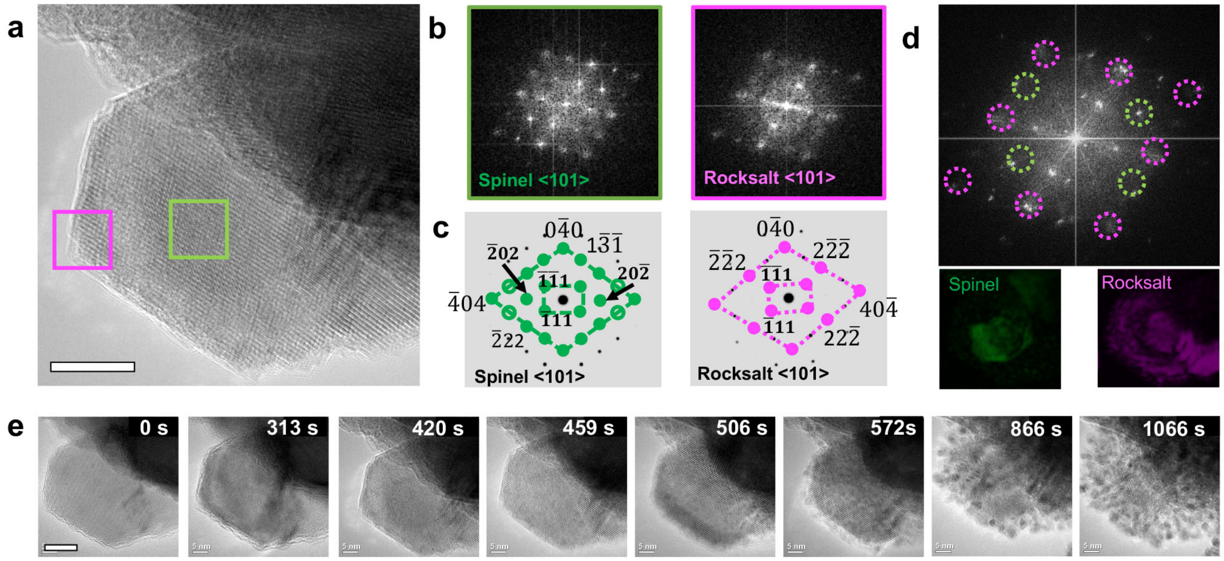

Supplementary Figure 8. (a) HRTEM image of a partially reacted L-ZFO (420 s). Scale bar: 10 nm. (b) FFTs obtains from the corresponding green and magenta squares shown in (a), respectively. (c) Simulated SAED patterns of spinel structure (green) and ordered rock-salt structure (magenta) along [101] zone axis, respectively. (d) FFT recorded along with HRTEM image shown in (a). Phase distribution of spinel (green) and ordered rock-salt (magenta) shown in below are obtained by selecting corresponding sets of spots in the FFT. (e) Time-sequenced HRTEM raw images showing morphology evolution of L-ZFO. Scale bar: 5 nm.

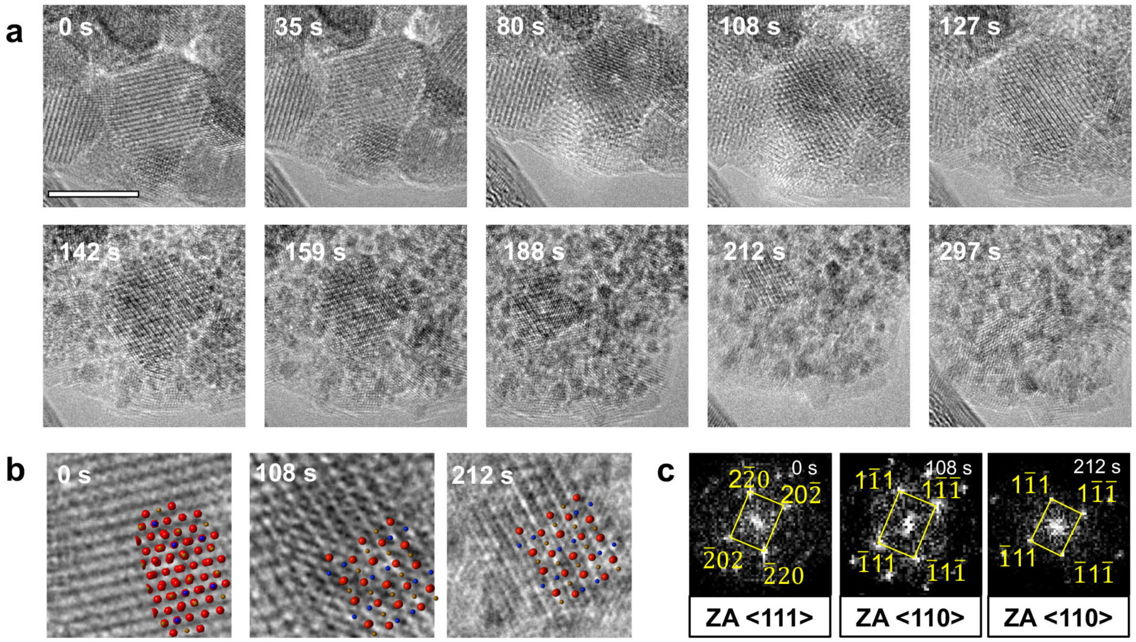

Supplementary Figure 9. (a) Time-sequenced HRTEM raw images showing morphology evolution of S-ZFO. Scale bar: 10 nm. (b) Enlarged HRTEM images illustrating crystallographic orientation. Overlaid models show the atomic structure of $ZnFe_2O_4$ projected along corresponding zone axis, respectively. (c) FFTs representing the phase information of particles shown in (b).

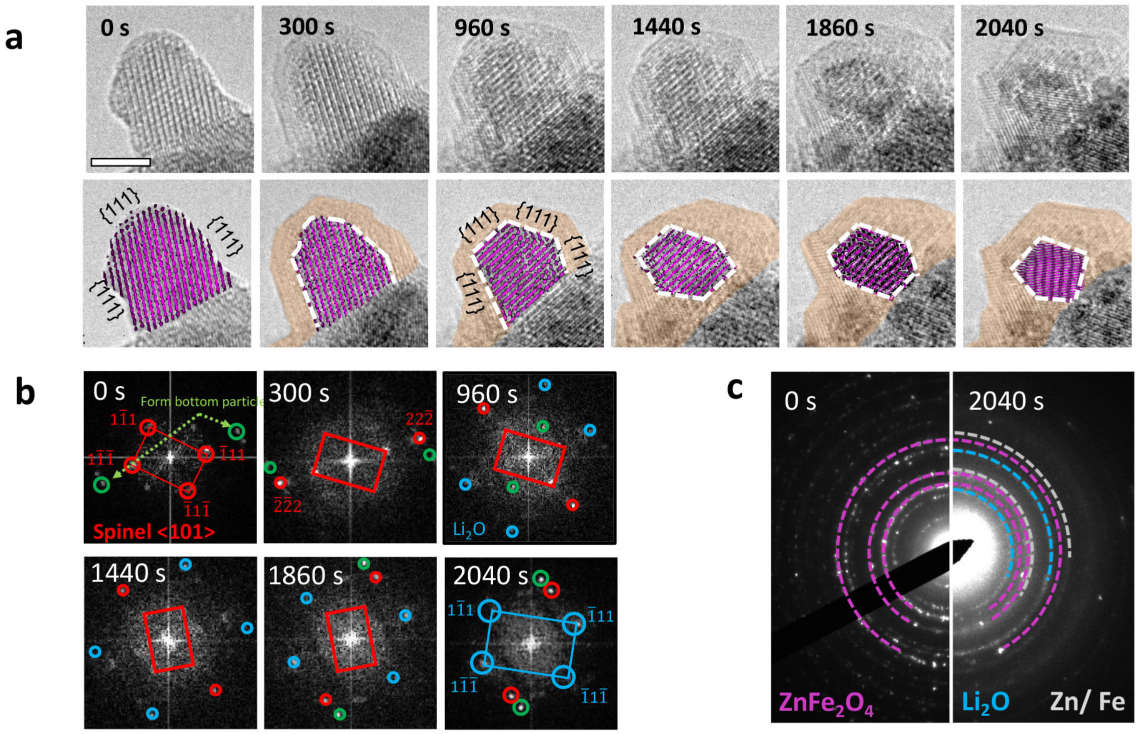

Supplementary Figure 10. (a) Time-sequenced HRTEM images showing morphology evolution of another S-ZFO. Spinel ZFO is shown in red and final product of conversion reaction is shown in orange. Scale bar: 5 nm. (b) Corresponding real time FFTs showing phase evolution as a function of reaction time. (c) SAED patterns of pristine (left) and final state (right) indicating the presence of unreacted spinel ZFO after 2040 s of lithiation.

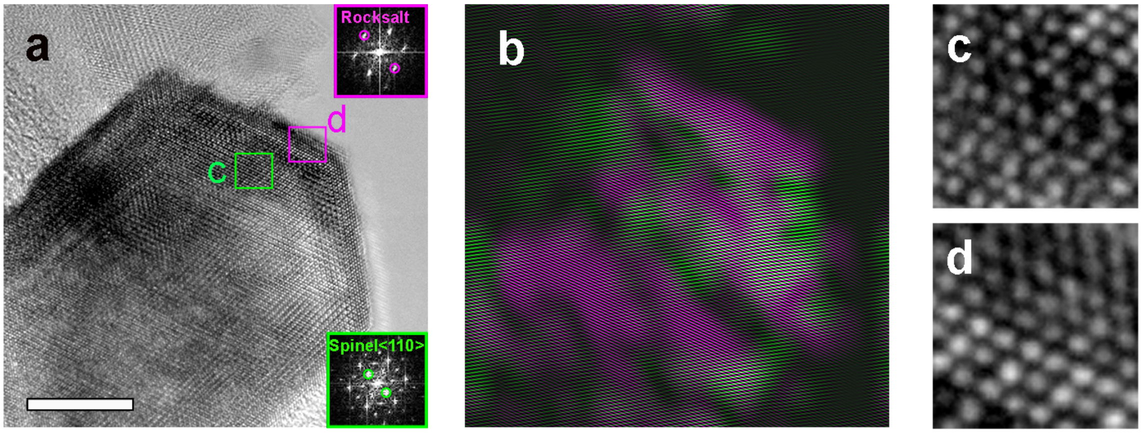

Supplementary Figure 11. (a) *Ex situ* HRTEM image of a partially lithiated L-ZFO, which has about less than 1 electron equivalent inserted (x < 1). Insets show the FFTs of the spinel (green) and rock-salt (magenta) structures along <110> zone axis. Scale bar: 10 nm. (b) Filtered images of (a) show the corresponding phase distribution using two sets of spinel and rock-salt FFTs. (c, d) The enlarged HRTEM images of square regions in (a) showing spinel and rock-salt structures, respectively.
