# Supplementary material for: Size-dependent kinetics during non-equilibrium lithiation of nano-sized zinc ferrite
Source: Nat Commun. 2019 Jan 9;10:93. doi: 10.1038/s41467-018-07831-5 (PMC6327060; doi:10.1038/s41467-018-07831-5)
Supplement: Supplementary file 2 — Description of Additional Supplementary Files [file 41467_2018_7831_MOESM2_ESM.pdf]

Supplementary Video Legends:

Supplementary Video 1: *In situ* electron diffraction pattern of small $ZnFe_2O_4$ during the entire lithiation process. The movie is accelerated by 8 times.

Supplementary Video 2: *In situ* electron diffraction pattern of large $ZnFe_2O_4$ during the entire lithiation process. The movie is accelerated by 13 times.

Supplementary Video 3: *In situ* HRTEM imaging of large $ZnFe_2O_4$ upon lithiation. The movie is accelerated by 71 times.

Supplementary Video 4: *In situ* HRTEM imaging of small $ZnFe_2O_4$ upon lithiation. The movie is accelerated by 20 times.
